# Integration of bulk RNA-seq and scRNA-seq reveals transcriptomic signatures associated with deep vein thrombosis

**DOI:** 10.3389/fgene.2025.1551879

**Published:** 2025-04-24

**Authors:** Bao-ze Pan, Ming-jun Jiang, Li-ming Deng, Jie Chen, Xian-peng Dai, Zi-xuan Wu, Zhi-he Deng, Dong-yang Luo, Yang-yi-jing Wang, Dan Ning, Guo-zuo Xiong, Guo-shan Bi

**Affiliations:** The Second Affiliated Hospital, Department of Vascular Surgery, Hengyang Medical School, University of South China, Hengyang, Hunan, China

**Keywords:** deep vein thrombosis, venous thromboembolism, WGCNA, bulk RNA-seq, ScRNA-seq, machine learning

## Abstract

**Background:**

Deep vein thrombosis (DVT) is a prevalent peripheral vascular disease. The intricate and multifaceted nature of the associated mechanisms hinders a comprehensive understanding of disease-relevant targets. This study aimed to identify and examine the most distinctive genes linked to DVT.

**Methods:**

In this study, the bulk RNA sequencing (bulk RNA-seq) analysis was conducted on whole blood samples from 11 DVT patients and six control groups. Topology analysis was performed using seven protein-protein interaction (PPI) network algorithms. The combination of weighted correlation network analysis (WGCNA) and clinical prediction models was employed to validate hub DEGs. Furthermore, single-cell RNA sequencing (scRNA-seq) was performed on peripheral blood samples from 3 DVT patients and three control groups to probe the cellular localization of target genes. Based on the same methodology as the internal test set, 12 DVT patients and six control groups were collected to construct an external test set and validated using machine learning (ML) algorithms and immunofluorescence (IF). Concurrently, the examination of the pathways in disparate cell populations was conducted on the basis of the CellChat pathway.

**Results:**

A total of 193 DEGs were identified in the internal test set. Additionally, a total of eight highly characteristic genes (including TLR1, TLR7, TLR8, CXCR4, DDX58, TNFSF10, FCGR1A and CD36) were identified by the PPI network algorithm. In accordance with the WGCNA model, the aforementioned genes were all situated within the blue core module, exhibiting a correlation coefficient of 0.84. The model demonstrated notable disparities in TLR8 (P = 0.018, AUC = 0.847), CXCR4 (P = 0.00088, AUC = 1.000), TNFSF10 (P = 0.00075, AUC = 0.958), and FCGR1A (P = 0.00022, AUC = 0.986). Furthermore, scRNA-seq demonstrated that B cells, T cells and monocytes play an active role in DVT. In the external validation set, CXCR4 was validated as a potential target by the ML algorithm and IF. In the context of the CellChat pathway, it indicated that MIF - (CD74 + CXCR4) plays a potential role.

**Conclusion:**

The findings of this study indicate that CXCR4 may serve as a potential genetic marker for DVT, with MIF - (CD74 + CXCR4) potentially implicated in the regulatory mechanisms underlying DVT.

## 1 Introduction

Deep vein thrombosis (DVT) is a common multifactorial disease with high morbidity and mortality, accounting for up to 40% of peripheral vascular disease. The incidence of DVT is also increasing due to an ageing population and the increased incidence of related comorbidities, including obesity, heart failure and cancer, with common chronic complications including post-thrombotic syndrome (PTS) (25%–38%) and venous ulcers (9.8%) (Duffett, 2022; Di Nisio et al., 2016; Bani-Hani et al., 2008; Kahn, 2010). Complications can occur immediately after acute DVT or months to years later. In clinical diagnosis, however, the diagnosis of DVT and its complications based on clinical presentation alone is unreliable due to the poor specificity of signs and symptoms (Boon et al., 2018). Venography is considered the “gold standard” for the diagnosis of DVT due to its invasiveness, high cost and technical characteristics (Khan et al., 2021). However, imaging is not recommended for every DVT patient due to the potential harms of the procedure, including radiation and contrast risks, as well as the associated medical costs, and is rarely used in daily practice (Khan et al., 2021; Koupenova et al., 2016). Furthermore, because of the susceptibility of the diagnostic process to interference from non-thrombotic conditions, including infection, surgery and tumours, the clinical use of non-invasive biomarkers with high specificity and sensitivity is lacking (Duffett, 2022; Kahn, 2010; Burgazli et al., 2013).

Bulk RNA sequencing (bulk RNA-seq) can reveal tissue-wide transcriptional expression profiles, but it masks differences between individual cells. In contrast, the emerging single-cell RNA sequencing (scRNA-seq) is a popular transcriptomics tool that reveals the expression profile of individual cells and complements traditional RNA-seq (Lu et al., 2023; Cheng et al., 2023; Nie et al., 2023). This not only explains the transcriptome heterogeneity of the cells, but also enables the identification of potential gene expression distributions, enabling personalized screening, diagnosis, treatment and prevention strategies for individual diseases (Nie et al., 2023; Li et al., 2023). Given this advantage, a large number of studies have focused on the identification of potential biomarkers of cardiovascular disease by integrating bulk RNA-seq and scRNA-seq assays, enabling precise stratification and identification of patients (Khan et al., 2024). In the bladder urothelial carcinoma (BLCA), hepatocellular carcinoma (HCC) and acute pancreatitis (AP), studies have been conducted in combination with bulk RNA-seq and scRNA-seq to characterize disease gene expression profiles, which have been biologically important in unraveling the pathogenesis of the diseases (Tan et al., 2023; Yu L. et al., 2022; Fang et al., 2022; Chi et al., 2023; Guo et al., 2023). As with the complex microenvironment of tumour diseases, there are also complex mechanisms of cell interaction in thrombotic diseases. However, there have been no similar reports in cases of DVT (Khan et al., 2024).

In peripheral vascular disease, as relevant tissues are not readily available, it is challenging to find large amounts of unbiased phenotypic data with transcriptome information to characterise the disease process (Cui et al., 2020). Concurrently, due to the powerful computational simulation capabilities of big data, the utilisation of machine learning (ML) methodologies to develop models has garnered increasing interest within the domain of medical research (Deo, 2015; Choi et al., 2020; Lo Vercio et al., 2020). The advantages of ML include the automated identification of information variables, the capture of non-linear relationships between variables, and the enhancement of diagnostic capabilities (Lee et al., 2021; Sultan et al., 2020; MacEachern and Forkert, 2021). In this context, ML has indicated great promise in various diagnostic and analytical modes, including least absolute shrinkage and selection operator (LASSO), logistic regression (LR), random forest (RF), artificial neural network (ANN), extreme gradient boosting (XGBoost), support vector machine-recursive feature elimination (SVM-RFE) and generalised linear model (GLM). With traditional statistical methods at their peak in terms of computational power, ML can explore new possibilities and unravel hidden disease-intrinsic relationships that can positively impact diagnosis and prognosis in the vascular field (Deo, 2015; MacEachern and Forkert, 2021; Rabbani et al., 2022).

**FIGURE 1:**
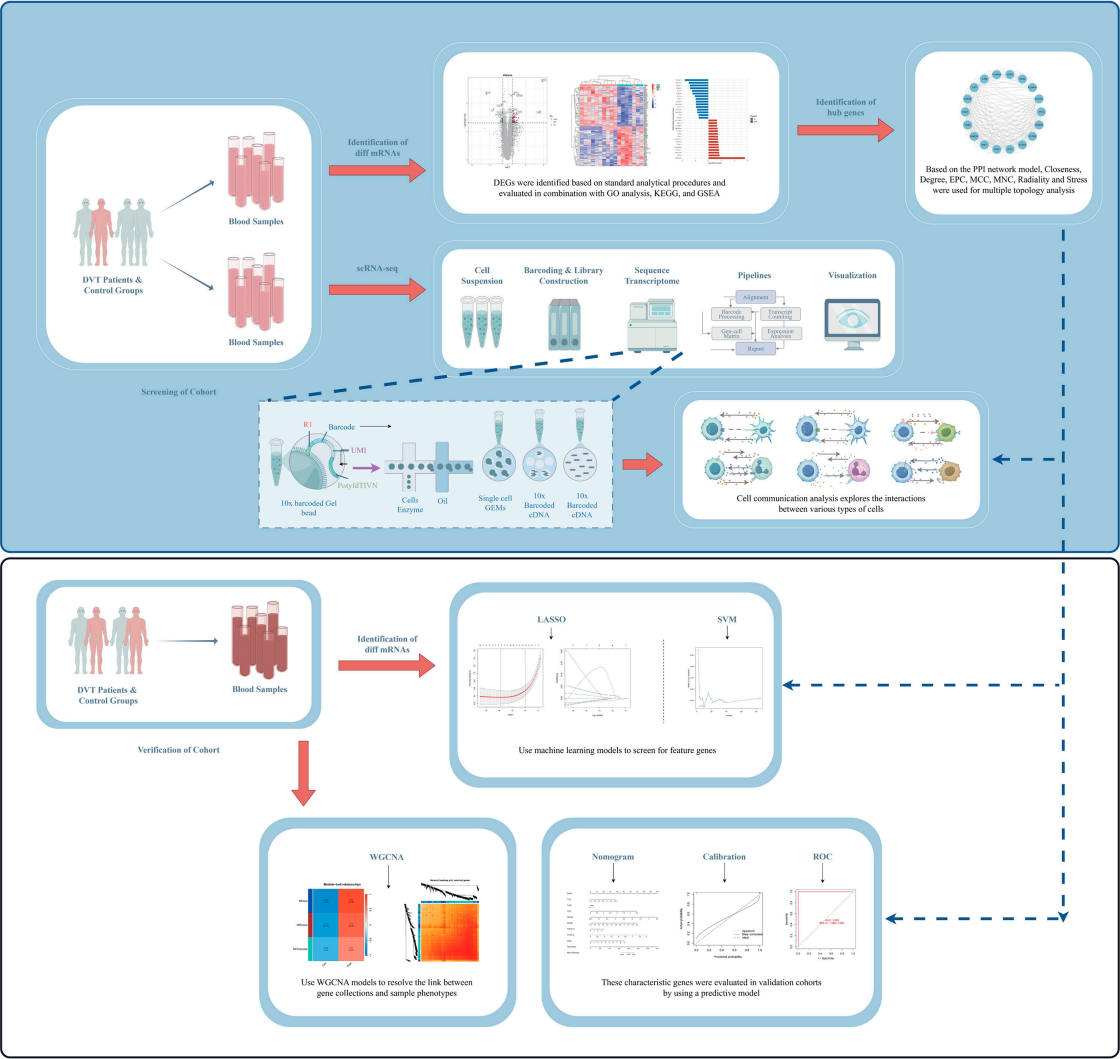
Workflow diagram presenting sample grouping and data processing, analysis and validation.

The objective of this study is to identify and investigate the transcriptome characteristics of DVT by integrating bulk RNA-seq and scRNA-seq. Based on the combined application of multiple models and algorithms, the disease-related susceptibility genes and corresponding targets were screened to comprehensively explore the complexity and intrinsic connectivity of regulatory sites in DVT (Figure 1).

## 2 Materials and methods

### 2.1 Sample collection

In accordance with the 2016 ISTH SSC classification guidelines, a total of 14 patients with unprovoked DVT diagnosed and treated by the Department of Vascular Surgery of the Second Affiliated Hospital of the University of South China, along with nine healthy controls from the physical examination center, were included in the study. The aforementioned samples were designated for inclusion in the internal test set between April 2023 and June 2023. Concurrently, 12 DVT patients and six control groups were enrolled as external validation sets using the same methodology. The period is from August 2023 to December 2023. The participants in both groups were matched according to gender, age and body mass index (BMI). The inclusion criteria were as follows: i) at least one test result (including ultrasonography or D-dimer test) supporting the diagnosis of DVT, ii) patients with a first episode of acute DVT, iii) not receiving anticoagulation, iv) age ≥ 18 years. The exclusion criteria are as follows: (i) antiphospholipid syndrome (APS), (ii) pregnancy or breastfeeding, (iii) infection or use of immunosuppressants within 2 weeks prior to enrolment, (iv) active or prior malignancy. At the time of admission, we collected the demographic and clinical data of the enrolled subjects, namely, gender, age, height, weight, medical history and laboratory biochemical indicators, and collected 6–8 mL of peripheral venous blood (DVT patients were drawn before treatment and healthy control groups were taken from the morning fasting blood), divided into two samples, and stored in ethylenediaminetetraacetic acid (EDTA) anticoagulant vessels. Centrifuge one sample for 5–10 minutesutes and then separate the plasma sample. We also isolated peripheral blood mononuclear cells (PBMCs) from another sample and added 500 ul of TRIzol (Invitrogen, United States) to adequately extract RNAs, and then stored in the −80 °C ultra-low temperature refrigerator (Thermo Fisher Scientific, United States). The study was conducted in accordance with the internationally recognized principles of the Declaration of Helsinki, and was reviewed and approved by the Hospital Ethics Committee of the Second Affiliated Hospital of the University of South China (approval number: 2022K091301). We also obtained handwritten informed consent from all subjects.

### 2.2 Determination of total genes

In accordance with the manufacturer’s protocol, total gene isolation and purification were conducted by using TRIzol on the samples of DVT patients (n = 11) and control groups (n = 6) previously collected. Also, the Fragment Analyzer (Agilent, United States) was used to detect the total gene amount, concentration, volume, RIN/RON value and 28S/18S of the sample. And then use the MGIEasy rRNA removal kit (MGI, China) to remove ribosomal rRNA from the total RNA according to the instructions. The library was constructed in accordance with the standard protocol of BGI Genomics Co. Ltd. (Shenzhen, China), and the data was analyzed on the Dr. Tom network platform (http://report.bgi.com). The raw data obtained from sequencing were subjected to filtration by using SOAPnuke (v1.5.6). Also, we used Ericscript (v0.5.5) for gene fusion assays, and rMATS (v3.2.5) for alternative splicing and differential alternative splicing assays. Then Gene expression quantification was performed with using RSEM (v1.3.1).

### 2.3 Identification of differentially expressed genes (DEGs)

The analysis of genes was conducted using the R packages edgeR (v3.32.1) and limma (v3.26.8) (log_2_ [FC] > 0.8 or log_2_ [FC] < −0.8). Concurrently, in order to gain a deeper understanding of the functional pathways of genes associated with phenotypic changes, based on hypergeometric tests, R packages clusterProfiler (v4.12.0) and org. Hs.e.g.,.db (v3.18.0) were used to perform Gene Ontology (GO) analysis and Kyoto Encyclopedia of Genes and Genomes (KEGG) enrichment analysis for DEGs. The results were compared with background genes to identify significant enrichment terms among candidate genes. Furthermore, in order to investigate the discrepancies in functions and associated pathways between disparate groups, the R package clusterProfiler was employed for gene set enrichment analysis (GSEA).

### 2.4 The multi-algorithm network analysis of the protein-protein interaction (PPI)

In order to gain insight into the biological significance of DEGs, we employed the STRING database (https://string-db.org/) to study their interactions at the protein level. Furthermore, Cytoscape (v3.10.0) was utilised for the purpose of visualisation. Concurrently, a multi-algorithm network analysis of cytoHubba (v0.1) was conducted to identify the top genes. The algorithms were selected for testing, including degree, closeness, maximum neighbourhood component (MNC), maximum clique centrality (MCC), edge percolated component (EPC), stress and radiality. The upset plot has been constructed to provide a comprehensive representation of the aforementioned results. To provide a comprehensive explanation of the biological pathways involved in the highly characteristic genes of the intersections, we conducted the GO analysis and KEGG enrichment analysis based on the same methodology.

### 2.5 Construction and validation of WGCNA

In order to identify highly synergistic gene sets, gene expression in the validation set was treated with R packages WGCNA (v1.72-5) and limma in order to describe gene association patterns between different samples. In order to construct a scale-free co-expression network based on a soft threshold, the adjacency matrix was used for all gene pairings. Subsequently, the adjacency matrix was converted into a topological overlap matrix (TOM), which enabled the weighted correlation between the two nodes and other nodes to be compared in order to represent the similarity of the nodes quantitatively. Concurrently, minModuleSize is set to 100 in accordance with the algorithm of the function blockwiseModules, which is employed for the purpose of dividing different co-expression network modules.

### 2.6 Construction and evaluation of the clinical predictive model

Based on the comprehensive expression of the external validation set, we employed the R package rms (v6.7-1) to construct the pertinent the nomogram for the highly characteristic genes that had been identified above, with a view to exploring their potential clinical value for the prediction of DVT risk. Additionally, the receiver operating characteristic (ROC) curve of the model was plotted by using the R package pROC (v1.18.5), and the discriminant power of the predictive model was assessed by measuring the area under the curve (AUC). The 95% confidence interval (CI) of the AUC values was calculated using the DeLong method to test for differences between AUCs. Subsequently, the calibration curve was plotted by using the lrm function to assess the degree of correspondence between the predicted and actual values. For each individual gene, ROC curves and differential boxplots were plotted separately in order to assess their efficacy.

### 2.7 Analysis of single-cell data

The nucleus suspension, with a concentration ranging from 700 to 1,200 per microliter as determined by Count Star, was loaded onto the Chromium Single Cell Controller (10X Genomics) to generate single-cell gel beads in emulsion (GEMs), using Gel Bead Kit (v3.1, 10X Genomics, 1,000,268) and Chromium Single Cell G Chip Kit (10X Genomics, 1,000,120). Concurrently, the captured cells underwent lysis, and the released RNA was barcoded through reverse transcription within individual GEMs. The library was constructed in accordance with the standard protocol established by BGI Genomics Co. Ltd., and the samples of DVT patients (n = 3) and control groups (n = 3) were subjected to analysis in accordance with the standard protocol. The cells were enumerated and filtered using R package Seurat (v3.0.2), and the initial 2000 hypervariable characteristic genes were identified by using Doubletdetection (v3.0). The function DimHeatmap is used to plot the principal component analysis (PCA) heatmap. Concurrently, the samples were performed nonlinear dimensionality reduction based on the t-distributed stochastic neighbour embedding (t-SNE) pathway, and the cell clustering results were visualised, with the R package SingleR (v2.6.0) subsequently used for the automatic annotation of cells. The default Wilcoxon rank sum test should be employed to identify significant DEGs between clusters (log_2_ [FC] > 1 or log_2_ [FC] < −1) and to ascertain cell types with highly characteristic gene localisation. Concurrently, to elucidate the cell-cell communication relationship and construct a cell-to-cell communication map, the R package CellChat (v1.6.1) was employed to analyse the cell-cell interaction.

### 2.8 Validation and analysis of ML models

In the external validation set, the R package limma was employed for the screening and analysis of DEGs (log_2_ [FC] > 1.5 or log_2_ [FC] < −1.5). In light of the aforementioned outcomes, a series of common ML algorithms, including LASSO and SVM-RFE, were executed using the R packages glmnet (v4.1-8), kernlab (v0.9-33) and caret (v6.0-94). ROC curves were plotted against the individually screened DEGs to determine statistical differences between the various model predictions and to rank their importance in order to account for their clinical relevance. In order to corroborate the findings of the PPI topology network analysis in the internal test set, the prediction outcomes of the aforementioned models were intersected, and the Venn diagram was constructed using the R package VennDiagram (v1.7.3). The prediction of human genetic variants falling within the normal range is facilitated by the Genotype-Tissue Expression (GTEx, https://gtexportal.org/home/). The prediction is derived from RNA-seq by Expectation-Maximization (RSEM) genes. The expression of human tissue for the crossover genes of the aforementioned validated models was visualised, and their underlying biological pathways explored by GSEA.

### 2.9 Immunofluorescence (IF) analysis

For histological analysis, the superficial veins of the lesions and normal parts of the lower limbs of DVT patients (n = 5) were surgically removed. The veins were then fixed with 4% paraformaldehyde for a period of 15 min at room temperature. The samples were permeabilised with 0.2% Triton X-100 (Beyotime, China) for 20 min, following which they were blocked with 5% bovine serum albumin (BSA) (Solarbio, China) for 1 h. The specimens were incubated with the primary antibody for 1 h and washed three times with PBST (PBS with 0.1% Tween-20). The primary antibodies employed in this study encompass the following: anti-CXCR4 (1:100, Proteintech, China). Following incubation with appropriate fluorophore-conjugated Cy3 goat anti-rabbit IgG (1:200, ABclonal, China) and 4′,6-diamidino-2-phenylindole (DAPI) (Thermo Fisher Scientific, United States), the slides were blocked with 20% glycerol (Servicebio, China) and then scanned and imaged using the inverted fluorescence microscope (MSHOT, China).

### 2.10 Analysis of cell-cell communication networks

In order to identify the ligand/receptor interaction involved in the characteristic genes, we conducted an analysis of the total reads of the same group of characteristic genes in the transcriptome. This was carried out using the R package CellChat and the web-based Explorer (http://www.cellchat.org/). The objective was to calculate the expression levels and quantify the network relationships from the perspectives of graph theory, pattern recognition and multiple learning.

### 2.11 Analysis of statistical results

The statistical analysis of all data was conducted using the R (v4.3.3) and SPSS (v23.0). The Kolmogorov-Smirnov (K-S) test was employed to ascertain whether the continuous data were normally distributed. In the case of data that are normally distributed, the t-test is employed and the results are expressed as a mean ± standard deviation. In the case of non-normally distributed data, Wilcoxon rank sum test was employed and the results are reported as the median of the quartiles. P-value <0.05 was considered statistical significance.

## 3 Results

### 3.1 Overview of total genes and DEGs

**FIGURE 2:**
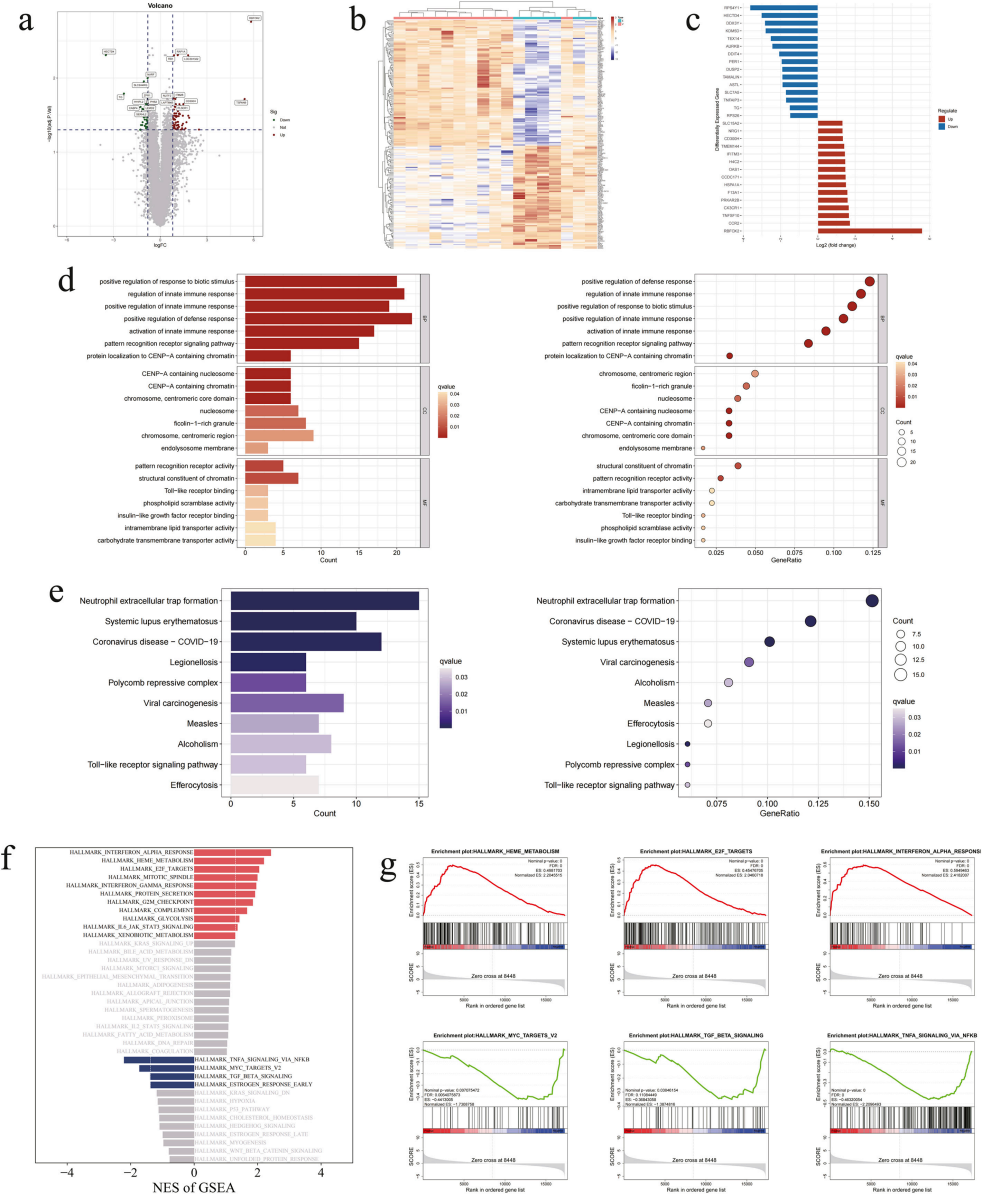
Initial identification, analysis and evaluation of DEGs. **(a)** Volcano plot of 193 DEGs. **(b)** Clustering heatmap of 193 DEGs. **(c)** Histogram of 193 DEGs. **(d)** GO analysis enrichment pathway and annotation diagram of 193 DEGs. **(e)** KEGG enrichment pathway and annotation diagram of 193 DEGs. **(f)** GSEA enriched pathway histogram of 193 DGEs. **(g)** GSEA enrichment plots of significant pathways.

In this study, according to the standard experimental procedure, a total of 17 samples and 120,455 genes were detected using the DNBSEQ platform after screening samples, sample extraction and high-throughput sequencing, with an average of 11.33 G data per sample. In order to identify statistically significant DEGs, a total of 193 distinct mRNAs (upregulated genes: 106, downregulated genes: 87) were obtained by utilising the R package edgeR and limma. Furthermore, to gain insight into the overall difference in gene expression profiles between the two groups, volcano plots were constructed for analysis (Figure 2a), and it was found that the distribution of DEGs was relatively balanced. The clustering heatmaps enables the visualisation of gene expression trends in a given sample based on colour changes (red: upregulated genes, blue: downregulated genes), thus facilitating the distinction between the differences between DVT patients and control groups (Figure 2b). Following the histogram to facilitate the visualisation of the aforementioned results in a quantitative manner (Figure 2c), RBFOX2, RPS4Y1 and HECTD4 were the most pronounced DEGs.

### 3.2 Analysis and evaluation of DEGs

In order to gain a comprehensive understanding of the biological pathways associated with the enrichment of DEGs, we conducted a GO analysis. This encompassed biological processes (BP), cellular components (CC) and molecular functions (MF). The resulting 178 terms pertain to the underlying biological mechanisms. Subsequent to the enrichment analysis, it was ascertained that the keyword most frequently occurring in DEGs within the BP category was positive regulation of response to biotic stimulus (GO: 0002833) and regulation of innate immune response (GO: 0045088). With regard to the context of CC, the DEGs are predominantly situated within the chromosome, centromeric core domain (GO: 0034506) and CENP-A containing nucleosome (GO: 0043505). In contrast, the enrichment pathway in MF is represented by pattern recognition receptor activity (GO: 0038187), structural constituent of chromatin (GO: 0030527) and Toll-like receptor binding (GO: 0035325) (Figure 2d). This indicates that the molecular mechanisms involved in the development of DVT are diverse and complex. To gain further insight into the pertinent biological processes, we conducted the KEGG analysis of the DEGs. Further analysis of the initial 15 pathways revealed that the formation of neutrophil extracellular traps (KEGG: hsa04613) constituted one of the most significant biological pathways in the development of DVT. Furthermore, 15 DEGs were identified as associated with this bioregulatory process (Figure 2e). In the concentrated display of GSEA, it can be inferred that the α-interferon pathway and TNF signalling pathway may be involved in the regulatory process of DVT (Figures 2f,g). This further identifies the most important biochemical and signal transduction pathways involved in the aforementioned candidate genes.

### 3.3 Evaluation of genes under PPI multi-network algorithm model

**FIGURE 3:**
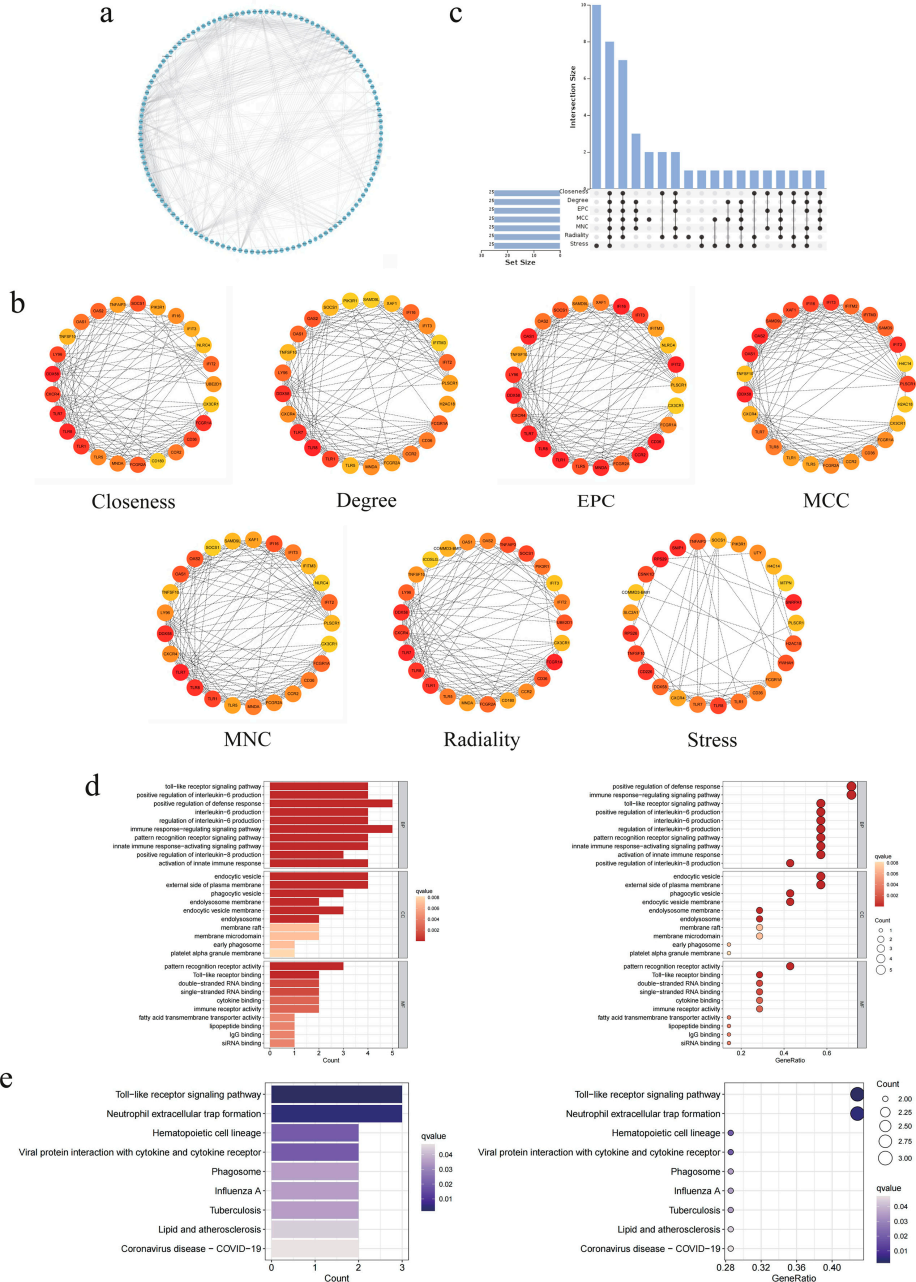
The hub DEGs were identified by the PPI network algorithm model. **(a)** PPI network diagram of 193 DEGs. **(b)** The top 15 DEGs were identified through multi-algorithm network analysis, including closeness, degree, EPC, MCC, MNC, radiality and stress, and color is redder with significance. **(c)** Upset plot of intersection with DEGs for seven algorithm network models. **(d)** GO analysis enrichment pathway and annotation diagram of eight highly characteristic genes. **(e)** KEGG enrichment pathway and annotation diagram of eight highly characteristic genes.

In order to achieve a more profound comprehension of the intricate interactions amongst the DEGs, it is necessary to elucidate their interrelations with signal transduction and cellular regulation. The aforementioned genes were initially identified in the STRING database. After following the implementation of the minimum interaction score of medium confidence (0.4), the PPI network was constructed and visualized using Cytoscape (Figure 3a). After comprehensive reference to coexpression and combined score, the results indicated that PLSCR1, DDX58, OAS1, XAF1 and FCGR2A were the most closely related core genes. Furthermore, topological analysis of multiple network algorithms demonstrated that TLR1, TLR7, TLR8, CXCR4, DDX58, TNFSF10, FCGR1A and CD36 were intersecting as highly characteristic genes with changes in node colour, thus providing a foundation for subsequent gene identification (Figures 3b,c). The results of the GO analyses and KEGG analyses for the aforementioned genes were analogous to those of the DEGs. Additionally, they were enriched in the immune response-regulating signaling pathway (GO: 0002764) and Toll-like receptor signaling pathway (KEGG: hsa04620), which provided a novel approach to the identification of biomarkers for DVT (Figures 3d,e).

### 3.4 Construction of WGCNA and identification of key module

**FIGURE 4:**
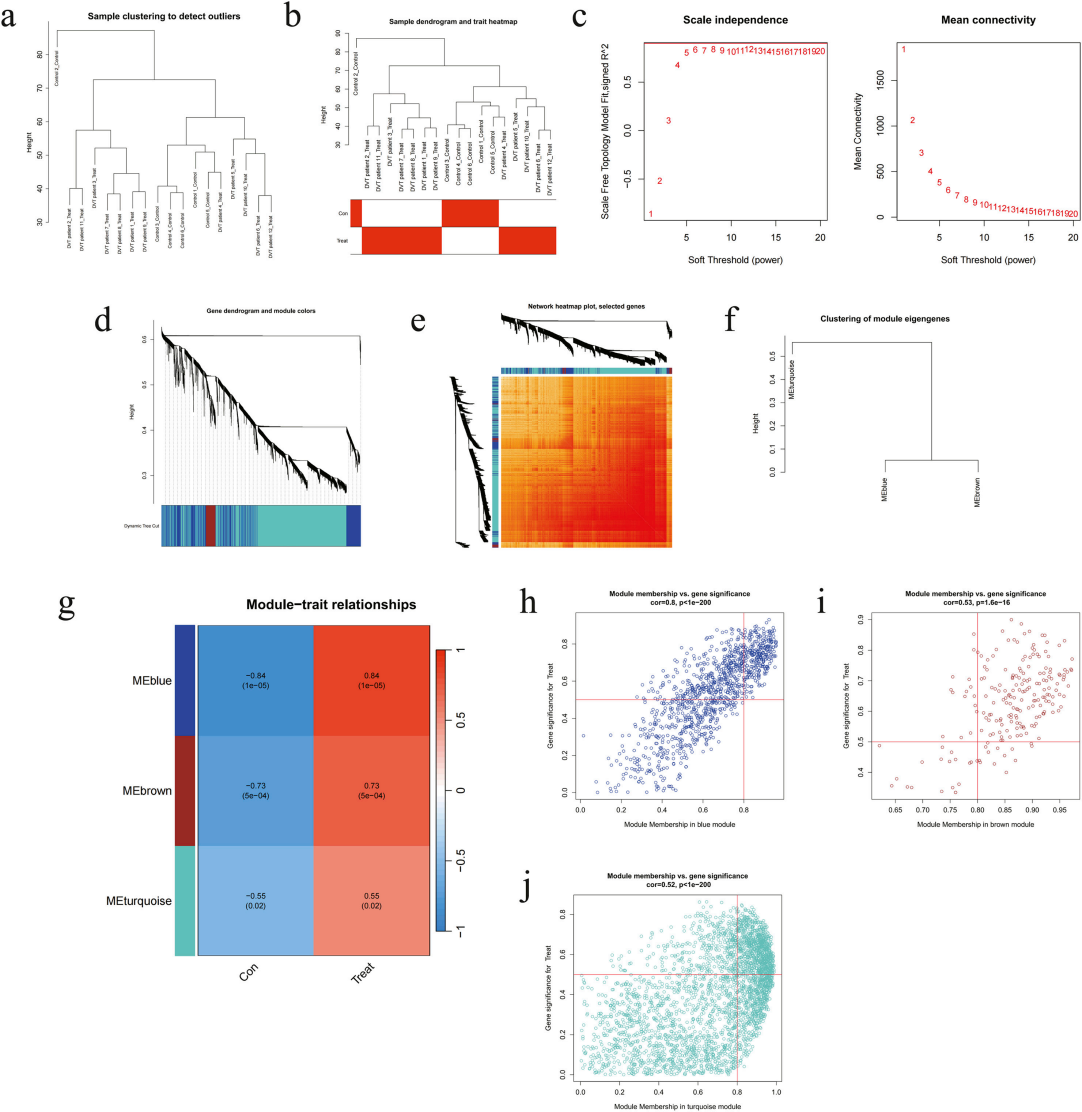
The hub DEGs were tested by WGCNA model in external validation set. **(a)** Hierarchical clustering of the system tree and corresponding sample information. **(b)** Sample system clustering tree and heatmap of related groups. **(c)** Network topology diagram of soft thresholds. **(d)** Cluster dendrogram of DEGs. **(e)** TOM heatmap. **(f)** Module clustering dendrogram. **(g)** Heatmap of correlations between modules and clinical traits (red: positive correlation, blue: negative correlation), and color is darker with correlation. **(h-j)** Scatter plots of DEGs and gene significance in different modules.

The Pearson correlation coefficient was employed for the purpose of grouping the samples. As illustrated in Figures 4a,b, a sample clustering tree and corresponding clinical feature heatmap were generated without the removal of outliers. From the scale-free soft threshold distribution map, it was determined that the optimal soft threshold was 4 (*R* ^2^ = 0.90) (Figure 4c). Following dynamic tree pruning and average hierarchical clustering, the grey modules indicated genes that were not suitable for these modules. Ultimately, three modules were identified (Figures 4d,e). To ascertain whether the aforementioned modules are associated with the clinical features of DVT, we proceeded to cluster the dendrograms of all DEGs based on differential measurements (1-TOM) (Figure 4f). Subsequently, we visualised the heat maps in order to reflect the correlation between the different modules and the size distribution (Figure 4g). To substantiate the genes of the key modules, a scatter plot was constructed to yield three modules in blue, brown and turquoise (Figures 4h–j). The above highly characteristic genes are all located in the blue module, with a correlation coefficient of 0.84, which lends further support to the accuracy of the screening results.

### 3.5 Establishment of clinical prediction model and assessment of genes

**FIGURE 5:**
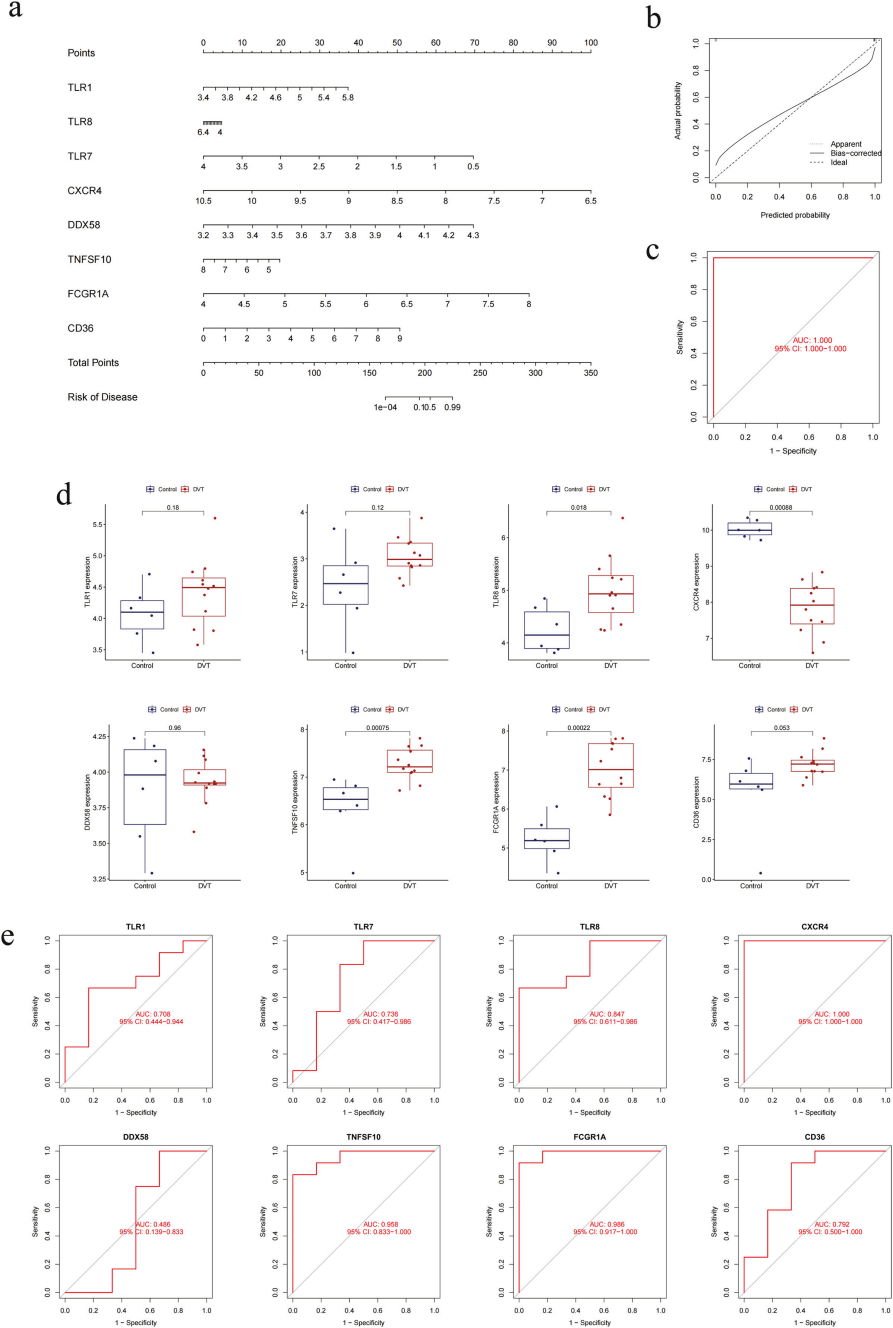
The hub DEGs are assessed by the clinical predictive model in external validation set. **(a)** Nomogram of eight highly characteristic genes. **(b)** Calibration curve of the clinical predictive model. **(c)** ROC curve of the clinical predictive model. **(d)** Boxplots of individual genes. **(e)** ROC curves of individual genes.

In order to evaluate the clinical utility of screening indicators, a nomogram was constructed to assess the predictive capacity of DVT (Figure 5a). This process commenced with the evaluation of each gene marker, followed by the drawing of a vertical line upwards to identify the corresponding dots. The total number of dots was then calculated by adding together the individual values. And the vertical line should be drawn down to indicate the DVT risk. The results demonstrated that the expression of CXCR4 and FCGR1A had the greatest capacity to assess disease risk. Furthermore, when the predicted probabilities were compared with the actual probabilities using calibration curves, it was found that the fit was also better, indicating a high degree of agreement (Figure 5b). The sensitivity and specificity of the model were evaluated using ROC curve analysis, which yielded an AUC of 1.000 (95% CI: 1.000–1.000). This result indicates that the model exhibited excellent classification performance and has high clinical application value (Figure 5c). To further evaluate the accuracy of the model, the boxplot of single-gene differences was constructed. This indicated that the eight genes selected demonstrated notable differences in the external validation set, including TLR8 (P = 0.018), CXCR4 (P = 0.00088), TNFSF10 (P = 0.00075), and FCGR1A (P = 0.00022) (Figure 5d). Furthermore, these genes are also significantly differentially expressed in thrombotic and non-thrombotic populations. Additionally, the ROC curves of the aforementioned single genes, including TLR8 (AUC = 0.847, 95% CI: 0.611–0.986), CXCR4 (AUC = 1.000, 95% CI: 1.000–1.000), TNFSF10 (AUC = 0.958, 95% CI: 0.833–1.000) and FCGR1A (AUC = 0.986, 95% CI: 0.917–1.000), were plotted, indicating that the model exhibits a high degree of reliability and that the aforementioned genes possess sufficient developmental value for disease prediction (Figure 5e).

### 3.6 Analysis of scRNA-seq to identify cell localization

**FIGURE 6:**
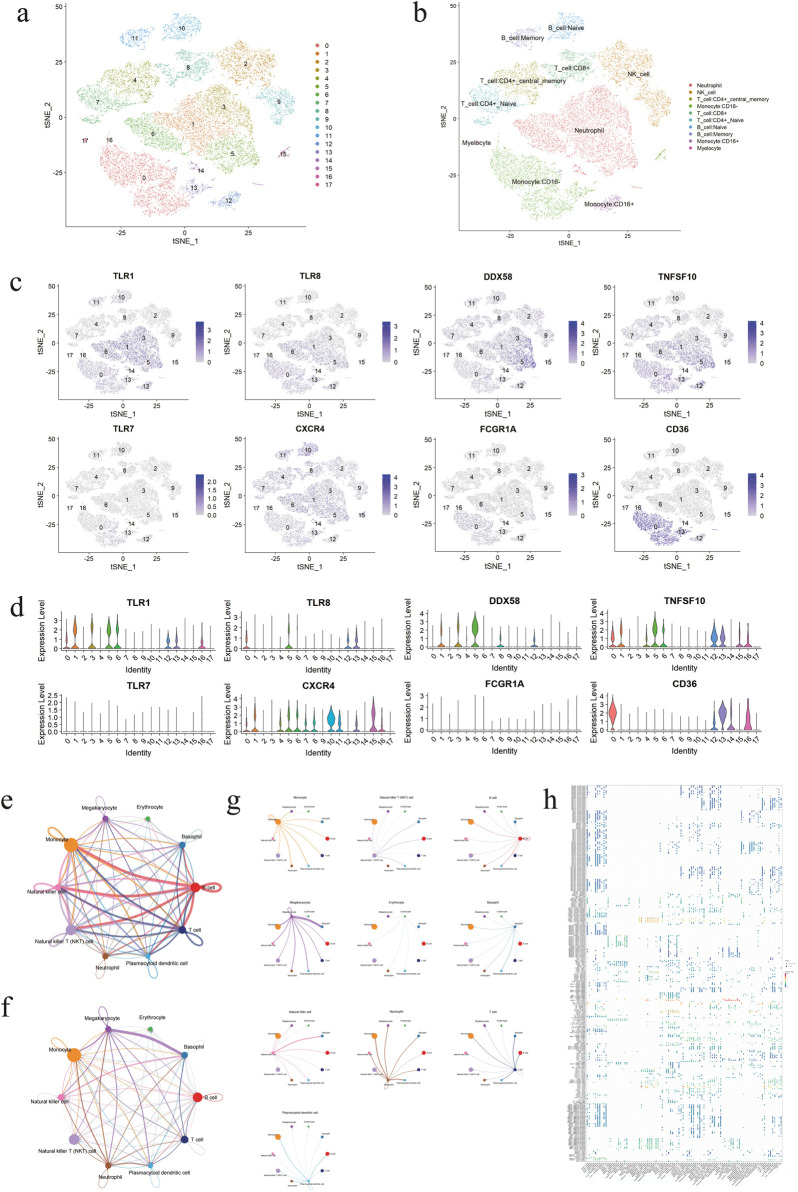
The cellular localization of genes were identified by single-cell analysis. **(a)** Diagram of t-SNE analysis of different 18 cell clusters. **(b)** Diagram of t-SNE analysis of 10 subcell types. **(c)** Feature plots of cell localization of eight highly characteristic genes. **(d)** Violin plots of cell localization of eight highly characteristic genes. **(e)** Diagram of cell-cell communication network based on the number of participating genes, with the thickness of lines representing the levels of the number. **(f)** Diagram of cell-cell communication network based on interaction weights, with the thickness of lines representing the levels of weights. **(g)** Diagrams of the communication network between a single cell population and other cells. **(h)** Bubble plot of the ligand/receptor pairs for major cell types.

In order to gain further insight into the behaviour of these genes at the cellular level during the pathogenesis of DVT, we conducted scRNA-seq analysis on samples obtained from DVT patients and control groups. Following the construction of the library, a total of 25,547 cells and 25,381 genes were identified through data comparison, filtering and statistical analysis. Following the implementation of quality control and visualisation procedures, with max_iter set to 1,000 and perplexity set to 30, t-SNE was able to effectively differentiate between the 18 different cell populations in the sample (Figure 6a). Preliminary inferences concerning heterogeneity and similarity were made on the basis of the two-dimension distribution of cell clusters. Concurrently, to ascertain the cell types with the most distinctive gene mapping, we conducted automated cell annotation using SingleR. This yielded 10 subcellular clusters: neutrophil, natural killer (NK) cell, central memory T cell (CD^4+^), monocyte (CD^16-^), T cell (CD^8+^), naive T cell (CD^4+^), naive B cell, memory B cell, monocyte (CD^16+^) and myelocyte (Figure 6b). Furthermore, the highly characteristic genes identified by the PPI multiplex network algorithm were located, and it was observed that CXCR4 was the most widely distributed, expressed in almost all cell clusters, and that TNFSF10 exhibited a similar expression pattern. Furthermore, CD36 is expressed almost exclusively in monocytes, whereas DDX58 is predominantly present in neutrophils. TLR1 and TLR8 are similarly expressed and are predominantly present in neutrophils, monocytes (CD^16-^) and monocytes (CD^16+^). The expression of TLR7 and FCGR1A is not significant (Figures 6c,d). Our results also demonstrate that the signalling pathways of immune cells exhibit a robust net number and weight/intensity of interactions (Figures 6e,f). Additionally, the above genes are extensively distributed in the exchange of information between B cells, monocytes, and NK cells (Figure 6g). Through a series of studies and quantitative comparisons, differential overexpression ligands and receptors for each cell group were likewise identified by CellChat, which helps elucidate the generality of cellular interactions. The aforementioned effects elucidate intricate signaling pathways that contribute to the development and progression of DVT, including MIF, TGFB1, NAMPT, LAGLS9 and CCL5. In the context of the aforementioned argument, MIF - (CD74 + CXCR4), MIF - (CD74 + CD44), TNFSF10 - (TNFRSF10B) and NAMPT - (ITGA5 + ITGB1) are also extensively implicated in signalling between B cells, T cells, monocytes and NK cells (Figure 6h), indicating potential therapeutic targets.

### 3.7 Validation of genes based on ML models and IF analysis

**FIGURE 7:**
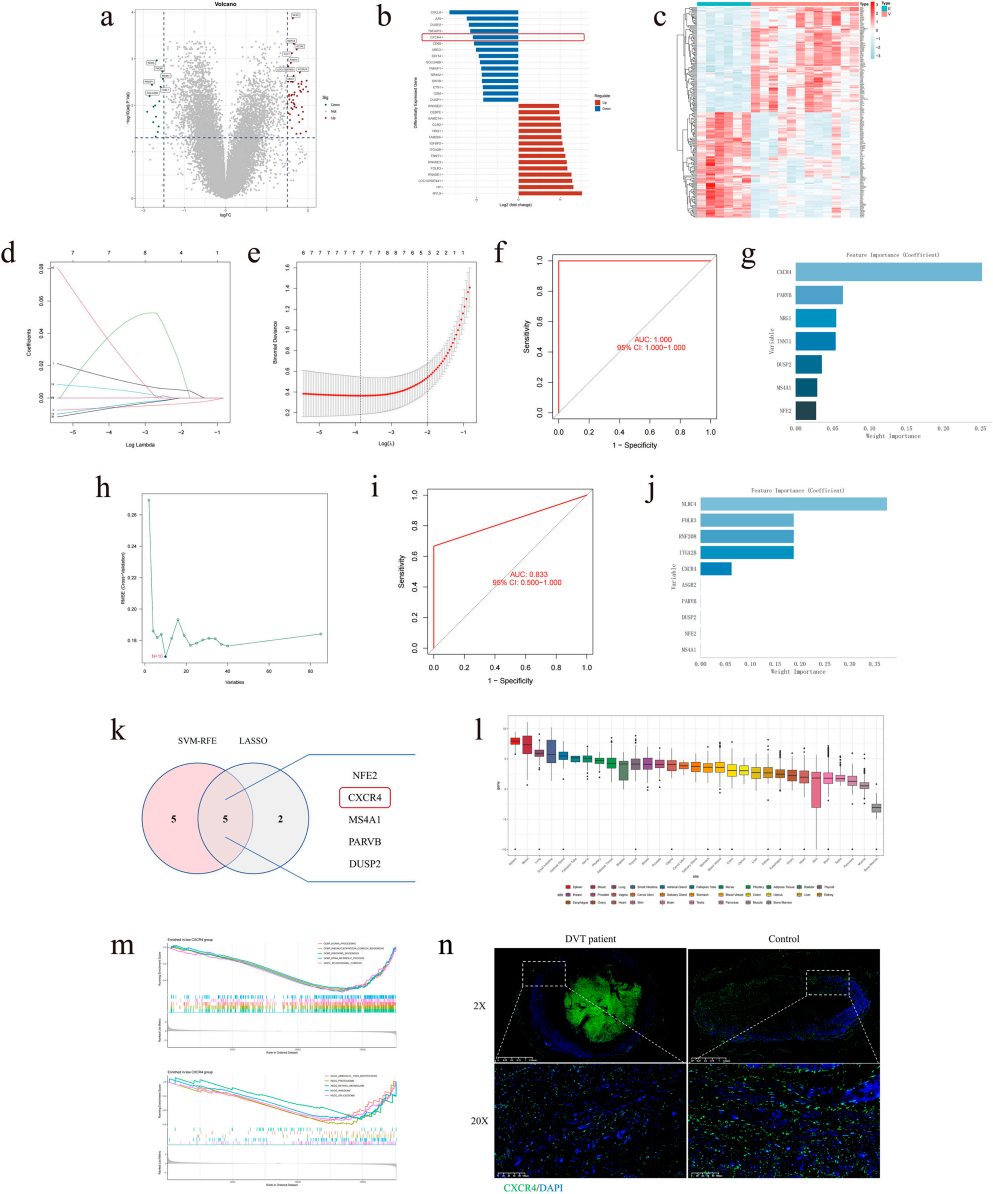
The hub DEGs were validated by ML models and IF in external validation set. **(a)** Volcano plot of 85 DEGs. **(b)** Histogram of 85 DEGs. **(c)** Clustering heatmap of 85 DEGs. **(d)** Regression coefficient path plot of LASSO model. **(e)** Cross-validation error plot of LASSO model. **(f)** ROC curve of LASSO model. **(g)** Feature importance ranking plot of LASSO model. **(h)** Feature curve of SVM-RFE model. **(i)** ROC curve of SVM-RFE model. **(j)** Feature importance ranking plot of SVM-RFE model. **(k)** Venn diagram of overlapping genes between ML models. **(l)** Boxplot of CXCR4 positioning predicted by GTEx. **(m)** GSEA enrichment plots for predicting significant pathways of CXCR4. **(n)** CXCR4 tissue expression was validated by IF (blue: DAPI, green: CXCR4).

In order to fully verify the organisational heterogeneity of CXCR4 between DVT patients and the control group, based on the results of differential analysis quantified by the external validation set, CXCR4 also demonstrated low expression as DEGs, exhibiting a significant trend between the two groups. These findings align with those of previous studies (Figures 7a–c). The LASSO regression analysis was employed to generate the penalty function as the variable coefficient of the regression model, resulting in the compression of the number of variables. This analysis revealed that among the 85 DEGs, seven eigencoefficients were non-zero (Figures 7d,e). The AUC of the model in the validation set was 1.000 (95% CI: 1.000–1.000) (Figure 7f), and CXCR4 also demonstrated the greatest significance in the feature importance ranking (Figure 7g). Similarly, in SVM-RFE, there were 10 characteristic variables to screen (Figure 7h), with a corresponding AUC of 0.833 (95% CI: 0.500–1.000) (Figure 7i), and CXCR4 was ranked fifth, demonstrating good specificity (Figure 7j). The Venn diagram illustrates that CXCR4 is the sole intersecting gene in the ML model described above (Figure 7k). Furthermore, CXCR4 was demonstrated to be the second most highly expressed gene in peripheral blood, following only the spleen, in the GTEx validation cohort. This provides a convenient reference point for clinical testing (Figure 7l). In the subsequent GSEA, CXCR4 was predicted to be involved in aminoacyl-tRNA synthetases (aaRSs) and was related to proteasomes and spliceosomes (Figure 7m). This provided a direction for further exploration of subsequent related mechanisms. Coincidentally, according to the IF results, the expression of CXCR4 was indeed significantly higher in peripheral blood cells than in vessel wall tissues. Compared to normal tissues, lesion tissues from DVT patients showed a significant downregulation trend (Figure 7n).

### 3.8 Analysis of MIF network pathways

**FIGURE 8:**
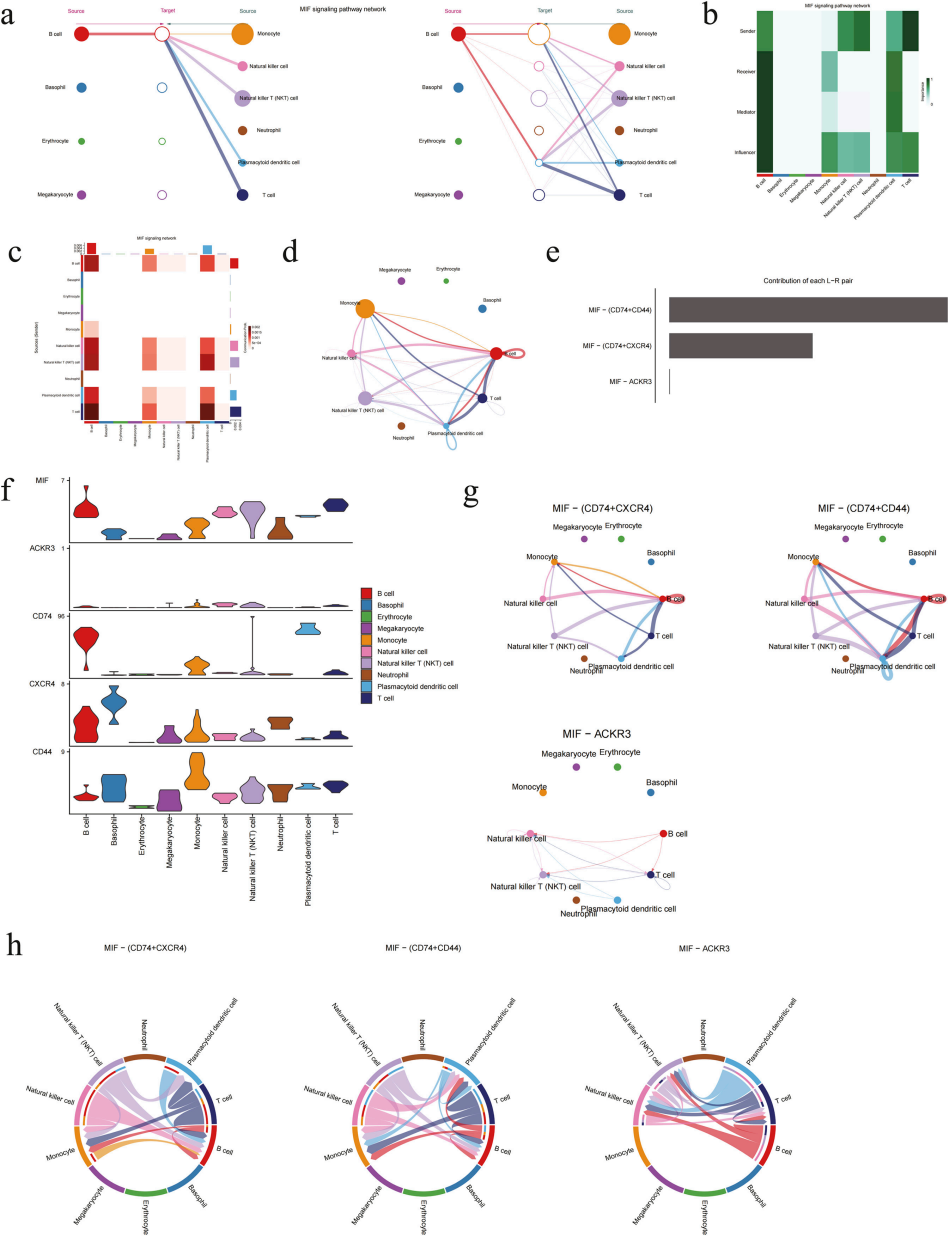
MIF signaling pathways were analyzed by CellChat. **(a)** Hierarchical plot of MIF pathways. **(b)** Heatmap of the incoming and outgoing signaling patterns, with color intensity representing relative strength of different signaling pathways. **(c)** Heatmap of communication differences across cell populations, with vertical axis representing senders and horizontal axis representing receivers, and color is darker with correlation. **(d)** Network diagram of cellular communication intensity, with the thickness of lines representing the levels of strength. **(e)** Ranking plot of the relative contribution of each ligand/receptor for MIF signaling pathway. **(f)** Violin plots of the expression pattern in MIF signaling pathway. **(g)** Network diagrams of different modes for MIF signaling pathway. **(h)** Chord diagrams of different modes for MIF signaling pathway.

In order to gain insight into the manner by which CXCR4 transmits signals and coordinates cell activities during the pathophysiology of DVT, a comprehensive comparison of the signal intensity of MIF - (CD74 + CXCR4) in different cell populations was conducted based on the CellChat method. Additionally, MIF - (CD74 + CD44) and MIF - (ACKR3) were included in the comparative study. As illustrated in Figures 8a,b, B cells exhibit aberrant activity within the MIF pathway described above, simultaneously engaging in cellular communication as senders, receivers, mediators and influencers. As with the preceding results, the interactions in monocytes, B cells, T cells and NK cells were significant in the heatmaps and network plots (Figures 8c,d). As the most prominent ligand/receptor pair in the MIF pathway (Figure 8e), CD74 and CXCR4 were expressed at higher levels in the aforementioned cells (Figure 8f). The network diagram and chord diagram also demonstrate that the biological exchange of MIF - (CD74 + CXCR4) between various cell populations is abundant (Figures 8g,h).

## 4 Discussion

In this study, bulk RNA-seq was employed to perform high-throughput analysis of whole blood samples from 11 DVT patients and six healthy control populations in the internal test set. The specificity of DEGs was evaluated and identified by combining the WGCNA algorithm and the clinical prediction model. Furthermore, calibration curves, boxplots and ROC curves were evaluated to ascertain the potential of these models to facilitate a more nuanced interpretation in clinical settings, thereby enhancing the precision of diagnostic and prognostic assessments. On this basis, scRNA-seq was performed on 3 DVT patients and three control groups to determine the cellular localisation and cellular communication pathway of the aforementioned high-characteristic genes. Furthermore, commonly used ML methods (including LASSO and SVM-RFE) were actively compared in the external validation set (n = 18), and the aforementioned results were verified by IF. This was done to comprehensively determine that CXCR4 can be used as a regulatory target related to DVT disease, with the aim of fully developing its diagnostic and predictive value. In light of the CellChat method, the expression of MIF - (CD74 + CXCR4) among disparate cell populations was examined in comparison with the other MIF pathways. This may provide a rationale for the development of effective strategies for the prevention and treatment of DVT.

CXCR4 is a G protein-coupled receptor that was initially identified in peripheral blood leukocytes (Chatterjee et al., 2014; Pozzobon et al., 2016). In addition to its established functions in haematopoiesis and immune responses, CXCR4 has also been demonstrated to play a pivotal role in neurogenesis, germ cell development and vascularisation (Pozzobon et al., 2016; Cui et al., 2013; Cheng et al., 2017; Bianchi and Mezzapelle, 2020; Kawaguchi et al., 2019). Furthermore, CXCR4 has been demonstrated to be indispensable for the homing, development and functionality of B cells (Chatterjee et al., 2014; Pozzobon et al., 2016). Our findings corroborate the long-standing notion of heightened CXCR4 expression in immune cells. Furthermore, they align with the validation outcomes of GTEx and IF. Its high expression during this process aligns with the cell localisation and targets identified in this study, suggesting its immense potential for clinical applications. Furthermore, CXCR4 has been identified as a prognostic marker for various types of cancer, including leukaemia, breast cancer and prostate cancer (Khare et al., 2021; Yu et al., 2023; Bao et al., 2023). During the process of cancer metastasis, the expression of CXCR4 can be increased, resulting in enhanced signalling pathways (Bao et al., 2023). This indicates its importance for the development and regulation of disease (Chatterjee et al., 2014; Bao et al., 2023). Currently, research on CXCR4 activity is concentrated on cytoplasmic-molecular interactions and cytoskeletal reorganization (Mousavi, 2020). In the field of cardiovascular disease, CXCR4 has been demonstrated to be overexpressed in abdominal aortic aneurysm (AAA) tissues. Nevertheless, the activation and expression patterns of CXCR4 transcriptional profiles in the various cell types involved in the development of DVT remain poorly understood. Similarly, CXCR4 primarily interacts with cells within the body’s microenvironment via the MIF - (CD74 + CXCR4) within the MIF signalling pathway (Ye et al., 2021). It is noteworthy that the above pathway can also promote B cell chemotaxis (Klasen et al., 2014). This finding is in alignment with the conclusions of the present study, which have led to the verification that B cells occupy a central regulatory position in DVT patients. MIF - (CD74 + CXCR4) is one of the more comprehensive research methods (Chen et al., 2024; Jankauskas et al., 2019; Schwartz et al., 2012; Lue et al., 2011). However, the current studies have only demonstrated an association between elevated plasma MIF levels and an increased risk of DVT in patients with spinal cord injury (Wu et al., 2019). While the precise interactions in their clinical expression remain elusive, the possibility that some patients exhibit a proclivity for disease sequelae based on the MIF pathway lends support to a pharmacogenomic approach to therapeutic intervention (Chen et al., 2024; Lue et al., 2011; Grieb et al., 2014; Zhang et al., 2024).

The advent of related omics technologies has led to the widespread utilisation of scRNA-seq in the investigation of individual differences and diversity amongst peripheral blood cells in patients (Tan et al., 2023). This biotechnological approach provides a comprehensive view of the dysregulation of DVT, facilitating the integration of cellular and molecular levels of analysis (Lu et al., 2023). Furthermore, based on WGCNA, PPI network algorithms and ML approaches, we conducted multi-angle and comprehensive algorithm analysis in external and internal datasets. This is achieved through the combination of scRNA-seq with traditional sequencing technologies, thereby facilitating a comprehensive investigation of the phenotypic and functional variation specific to DVT patients. The identification of differential cell subsets associated with DVT and the ligand/receptor alterations associated with MIF can facilitate the elucidation of the underlying mechanisms that drive DVT disease. This integrative approach has the potential to reveal novel regulatory networks that may be involved in the pathogenesis of DVT. Furthermore, scRNA-seq serves to validate the functional role of genes, thereby enabling an exploration of their ability to determine the causal relationship between identified characteristic cell perturbations, signalling pathways, and disease progression (Lu et al., 2023; Xue et al., 2023).

Over the past several years, there has been a notable increase in the utilisation of ML technology in the healthcare sector. This is largely due to the fact that ML is employed extensively in the prediction of novel disease targets, largely as a consequence of its exemplary performance in clinical diagnosis (Deo, 2015; Wei et al., 2024). Despite the availability of clinical rating scales for the prediction of DVT risk, the study by Mooney et al. has demonstrated that ML outperforms traditional scoring methods in terms of prediction accuracy (Coombs et al., 2022; Jin et al., 2022; Yu T. et al., 2022). ML methods have the potential to enhance the precision of predictive outcomes by elucidating the complex interrelationships between risk factors. This growth can be attributed to the emergence of larger data sets, electronic medical records, and more sophisticated application processes (Deo, 2015; Hollon et al., 2018). ML models are capable of automatically identifying the most predictive features, in contrast to the manual identification of disease features. Furthermore, this information can be generalised to new patient populations (Hollon et al., 2018). In our study, LASSO is employed to enhance the predictive accuracy and comprehensibility of statistical models. This is due to the fact that the LASSO is a regression technique for variable selection and regularisation, and it is extensively utilised in the medical field (Wang et al., 2022). SVM-RFE, a supervised ML technique that is extensively utilised in classification and regression, is also capable of identifying the optimal combination of variables to ensure maximum model performance (Deo, 2015). CXCR4 demonstrates both specificity and sensitivity across a range of data sets, as evidenced by the utilisation of the aforementioned algorithm model for verification. This provides a promising avenue for subsequent research, which may facilitate the quantification of the individualised risk of DVT and inform clinical decisions regarding thromboprophylaxis (Yu T. et al., 2022; Xue et al., 2021). To the best of our knowledge, there are currently no studies that have made a comparison between the predictions of DVT risk and the identification of potential biological targets that are associated with DVT.

The principal benefit of this study is the integrated analysis of bulk RNA-seq and scRNA-seq, which permits a comprehensive examination of cell-cell interactions at the system level. Subsequent studies should employ integrated ML algorithms to validate diagnostic biomarkers with significant categorical effects. This will facilitate the creation of multiplex assays, the assessment of disease modulation processes, the aiding of clinical diagnosis, the determination of time windows, and the guidance of clinical decision-making. Nevertheless, it is important to acknowledge the potential limitations of our research. Firstly, with regard to the clinical prediction model, the instability and uncertainty in the judgement of clinical decision-making is a consequence of the small sample size. In order to enhance the clinical applicability of this study to DVT patients exhibiting disparate molecular characteristics, it is imperative to incorporate a more expansive cohort of participants, thereby facilitating a more comprehensive evaluation of the prognostic and therapeutic implications. Furthermore, the latest model interpretation techniques do not consider the interdependencies between features, and correlation statistical analysis is unable to provide an accurate representation of the underlying pathophysiological processes, potentially introducing significant biases. Consequently, there is a need for further research to develop a comprehensive profile of peripheral blood changes in DVT patients.

## Data Availability

The raw data supporting the conclusions of this article will be made available by the authors, without undue reservation.
