# Supplementary material for: Integration of bulk RNA-seq and scRNA-seq reveals transcriptomic signatures associated with deep vein thrombosis
Source: Front Genet. 2025 Apr 24;16:1551879. doi: 10.3389/fgene.2025.1551879 (PMC12060172; doi:10.3389/fgene.2025.1551879)
Supplement: Supplementary file 1 [file DataSheet2.pdf]

Table2. Characteristics of DVT patients and control groups for scRNA-seq

Name	DVT patient_1	DVT patient_2	DVT patient_3	Control_1	Control_2	Control_3
Sex	male	male	female	male	male	female
Age	63	72	67	73	70	56
Admission Time	2023/4/9	2023/4/19	2023/5/24	2023/4/28	2023/5/9	2023/5/14
BMI	25	29	27	30	29	26
Smoking	No	No	No	No	No	Yes
Alcohol Consumption	No	Yes	No	Yes	No	Yes
Coronary Heart Disease	No	No	Yes	No	No	No
Diabetes	No	No	No	No	No	Yes
Hypertension	No	Yes	Yes	Yes	No	Yes
Hyperlipidemia	No	No	No	No	No	No
Total Cholesterol (mmol/L)	5.32	5.11	5.14	6.67	5.85	5.31
Triglycerides (mmol/L)	1.83	1.03	1.93	5.18	1.97	1.53
High Density Lipoprotein (HDL) (mmol/L)	1.04	1.37	1.71	1.16	1.1	1.45
Low Density Lipoprotein (LDL) (mmol/L)	3.29	2.76	2.76	3.76	3.7	3.04
