# Supplementary material for: Integration of bulk RNA-seq and scRNA-seq reveals transcriptomic signatures associated with deep vein thrombosis
Source: Front Genet. 2025 Apr 24;16:1551879. doi: 10.3389/fgene.2025.1551879 (PMC12060172; doi:10.3389/fgene.2025.1551879)
Supplement: Supplementary file 2 [file DataSheet1.pdf]

Table1. Characteristics of DVT patients and control groups for bulk RNA-seq

Variables	DVT patient (n=23)	Control group (n=12)	p-Value
Male, n (%)	12, (52.17)	4, (33.33)	0.4759
Age, years	61.17±3.017	58.58±1.540	0.5553
BMI	24.13±0.6482	22.50±0.8118	0.1382
**Personal History, n (%)**			
Smoking	2, (8.695)	2, (16.67)	0.5941
Alcohol Consumption	3, (13.04)	1, (8.333)	0.9999
Coronary Heart Disease	1, (4.347)	1, (8.333)	0.9999
Diabetes	5, (21.73)	1, (8.333)	0.6399
Hypertension	5, (21.73)	2, (16.67)	0.9999
Hyperlipidemia	2, (8.695)	0, (0.000)	0.5361
**Hematological Indicators**			
Total Cholesterol (mmol/L)	5.307±0.2680	5.689±0.3059	0.3833
Triglycerides (mmol/L)	2.012±0.3324	1.853±0.4806	0.7845
High Density Lipoprotein (HDL) (mmol/L)	1.234±0.06730	1.524±0.09876	0.0188
Low Density Lipoprotein (LDL) (mmol/L)	2.928±0.1783	3.164±0.2742	0.4601
